# Editorial for the Special Issue: “Hydrothermal Synthesis of Nanoparticles”

**DOI:** 10.3390/nano13091463

**Published:** 2023-04-25

**Authors:** Juan Carlos Rendón-Angeles, Gimyeong Seong

**Affiliations:** 1 Center for Research and Advanced Studies of the National Polytechnic Institute, Mexico City 25900, Mexico; 2 Department of Environmental and Energy Engineering, The University of Suwon, 17, Wauan-Gil, Hwaseong-si 18323, Gyeonggi-do, Republic of Korea; 3 New Industry Creation Hatchery Center, Tohoku University, 6-6-10 Aoba, Aramaki, Aoba-Ku, Sendai 980-8579, Japan

Research and development in materials science has improved tremendously over the past few decades, resulting in benefits to the quality of life of people worldwide. In parallel, nanoparticle synthesis has emerged as a rapidly growing field of nanotechnology research. Nanoparticles (NPs), including organic and inorganic materials (such as metals, metal oxides, polymers, etc.), are tiny finite solids with at least one geometrical dimension below 100 nm. According to their size, NPs possess specific physical, chemical, optical, catalytic, and electronic properties compared to their large-sized parent bulk materials. Therefore, nanoparticles have various applications in catalysis, medicine, pharmaceutical, agriculture, and electronics, among other specific research fields. These applications are microstructurally dependent on nanoparticles’ large surface-to-volume ratio, which allows them to interact rapidly with other particles of larger size.

Since natural NPs were discovered, the synthesis of NPs has become a primary research subject. Hitherto, the techniques proposed for the preparation of NPs are based on physical and chemical principles. Physical methods are founded on a top-down methodology, grinding being the most representative among these. It involves the dimensional reduction of solid matter to a nanoscale level. Other advanced techniques based on the same principle involve sputtering, laser ablation, electrospraying, and electron beam evaporation. On the contrary, the chemical methods developed for NP preparation differ in the technical philosophy applied; these methods are established on a bottom-up methodology that comprises the controlled manoeuvre of the atoms or molecules employed to form the NPs. Chemical processing techniques provide suitable reaction conditions for the production of NPs with specific microstructural aspects (e.g., size and morphology), compositions, and crystalline structures, which are the significant technological advantages of these processing methods. Nowadays, solution-based techniques such as coprecipitation, sol–gel, membrane-based, sonochemical, pyrolysis, vapour deposition, microwave-assisted, ion exchange, and combustion techniques have been widely employed to produce a wide variety of NPs.

Hydrothermal synthesis is a well-known technique used by various research groups worldwide to prepare NPs under subcritical or supercritical water conditions. This technique follows the bottom-up approach and has recently been used as a potential route for preparing a broad type of inorganic NPs. In particular, the heterogeneous chemical reactions that trigger the manipulation of hydrolysed atomic species in water can be achieved in a temperature range of 100–500 °C and high pressures varying from 0.1 to 22.5 MPa or more. However, some researchers also correlate the “hydrothermal” term with aqueous systems under reaction below 100 °C. In the last two decades, particular efforts have been made to provide faster nucleation kinetics and limited particle growth, resulting in the preparation of particles with specific morphologies and nanosized dimensions.

Additionally, the excellent properties of water have made it the research subject of new research groups for use in the production of nanoparticles and microparticles in a wide area of investigation encompassing various production scenarios, from laboratory-scale (basic chemical reaction studies) to pilot plant-scale production. Furthermore, the prepared nanomaterials’ chemical compositions are controlled in the hydrothermal reaction media through a liquid phase or multiphase chemical reactions. Hence, these advantages provide an efficient processing technology to produce metal, oxide, and hybrid NPs.

This Special Issue, titled “Hydrothermal Synthesis of Nanoparticles”, compiles the efforts of various groups of researchers worldwide and aims to communicate the recent advances in this research field. Nine original research papers focusing on synthesising different inorganic materials for heat transfer, biomedical, photocatalysis, scintillator, and photoluminescence applications are included. Furthermore, one review presents the state-of-the-art on SiC oxidation under dry and hydrothermal conditions. The following paragraphs give an overview highlighting the contributions of each published article.

The effort to control heat transfer is essential in the industry because of the high cost implied with the waste of heating energy. Hence, heat management has become a subject of concern in the equipment used in the industry, such as heat exchangers, automobiles, high-voltage transformers, refrigerators, electronic circuits, nuclear systems, solar energy harvesting plants, and desalinisation machines. Alam et al. [1] studied an innovative methodology for preparing nanofluids with thermal insulating properties. Zinc oxide nanorods particles were proposed as an insulating material, and the preparation of ZnO nanorods was successfully achieved at a low temperature of 170 °C for 6 h. A surfactant agent (polyvinylpyrrolidone, PVP) controlled the particle morphology and size. Heat transfer coefficient measurements were conducted to determine the nanofluid’s efficiency. The results revealed that the laminar nanofluids constituted by 0.3 vol% of ZnO nanorods and ethylene glycol had a good heat transfer coefficient. These results open a new possibility for expanding this technology for heat management applications at the industrial level.

Various studies have been carried out in the last two decades to prepare biomaterials with similar chemical properties to biological calcium hydroxyapatite (HAp). The incorporation of silicon in the hexagonal structure of HAp has become a research subject, in which the veracity of the Si^4+^ substitution is under discussion to some extent. Matamoros-Veloza et al. [2] investigated the preparation of substituted silicon hydroxyapatite (Si-HAp) nanoparticles via the microwave-assisted hydrothermal process and employing C_4_H_13_NO_5_Si_2_ (TMAS) as a Si^4+^ precursor. Si^4+^ uptake improved by saturating the hydrothermal media with TMAS. The highest content of Si^4+^ obtained at 150 °C for 1 h was 12.16 mol%. Excess Si^4+^ also triggered the formation of Si-HAp agglomerates, constituted by fine nanorod particles of 32 nm.

Furthermore, the fine particles exhibited a 3D hierarchical self-assembly process favoured by the Si^4+^ ions in aqueous media coupled with rapid kinetic reaction conditions assisted by microwave heating. Additionally, the functionality of HAp NPs as potential catalyser agents was proposed by Nakagiri et al. [3]. The catalytic properties of calcium hydroxyapatite NPs were investigated as a function of the Ca/P molar ratio of the HAp. HAp NPs with various Ca/P ratios, between 1.54 and 1.72, were prepared under hydrothermal conditions at 110 °C for 16 h. The catalyst containing nonstoichiometric HAp NPs and a mixture of SiO_2_/P_2_O_5_ exhibited remarkable catalytic behaviour for converting 1,6 hexanediol. The dependence on the Ca/P molar ratio in preparing unsaturated and cycloalkane alcohols was demonstrated for the first time. The particle size and the compositional variation inherited from the HAp NPs resulted in higher yields and sufficient selectivity than that reported in previous studies related to the catalytic behaviour of stoichiometric HAp particles.

Perovskite materials have been prepared under a wide range of hydrothermal conditions; thermodynamically, oxides are highly stable under alkaline hydrothermal media. In this research field, Burnett et al. [4] have presented an exciting study to optimise the chemical reactivity of the raw materials Ta_2_O_5_ and IrCl_3_ to establish chemical equilibrium to produce iridium containing NaTaO_3_ in a single step. A non-common alkaline medium constituted by 10 M NaOH in 40 vol% H_2_O_2_ heated at 240 °C was used. This aqueous medium is highly reactive to produce cube-shaped NaTa_1-x_Ir_x_O_3_ crystallites around 100 nm in size. Interestingly, the perovskite nanoparticles underwent a heat treatment that caused the reduction of Ir^4+^, partially substituting Ta^5+^ in the perovskite structure.

Consequently, the oxide particles were covered by the Ir^0^. A ten-fold capability on hydrogen production, determined using a water–methanol solution, was observed on the NaTa_1-x_Ir_x_O_3_ crystallites rather than the parent NaTaO_3_. A significant proportion of H_2_ was ascribed to visible light absorption; therefore, these materials have the potential to be employed in the water-splitting catalyst medium for H_2_ generation.

Nanoparticle dispersibility is an essential property for preparing organic solvent scintillators that can be improved via the morphological modification of the particles. In this regard, the subcritical and supercritical hydrothermal conditions provide an adequate reaction environment to maximise the crystallisation of highly monodispersed nanoparticles. The study conducted by Watanabe et al. [5] aimed to improve the dispersibility of ZrO_2_ NPs in toluene because it is well-known that the double-beta decay of 96Zr has a high Q value of 3350 keV; therefore, this oxide can play the role of an isotope for 0*νββ* detection via radioluminescence spectroscopy. According to the research results, the organic 6-phenyl hexanoic acid part controlled the NP’s growth via surface modification and was established as a function of the temperature. At a ZrO_2_ NPs content of 0.33 wt%, prepared under subcritical conditions (300 °C for 10 min) and dispersed in toluene with 1,4-bis(5-phenyl-2-oxazolyl) benzene and 2,5-diphenyl oxazole, the as-prepared liquid scintillator had the maximum X-ray-induced radioluminescence response. It was concluded that only a small amount (0.0092 wt%) of ^96^Zr isotope is required to prepare a liquid scintillator for potential usage in radiotherapy.

Fundamental studies based on parametric optimisation analyses correlating hydrothermal media selection, solvent pH, the chemical stability of the target crystalline phase, and precursor reactivity, among other things, are essential to determine the feasible morphological tuning of NPs to be prepared under hydrothermal conditions. Zheng et al. [6] applied this methodology for synthesising Eu(OH)_3_ submicron particles and nanoparticles. Eu(OH)_3_ was chemically stable in the pH range of 7.26–12; in acidic conditions, the dominating phase was Eu_2_(OH)_5_NO_3_•2H_2_O in the Eu_2_O_3_-NO_3_-NH_4_OH-H_2_O system. A remarkable difference in the particle morphologies occurred in the experiments conducted between 80 and 240 °C for 24 h. Eu(OH)_3_ produced at pH values over 9 exhibited nanorods, nanotubes, and euhedral shapes. The differences in the solute’s saturation level, which is temperature-dependent, trigger the formation of a large molar volume of the nucleus that limits the growth of Eu(OH)_3_ crystals at temperatures over 200 °C. Likewise, the low solubility of Eu(OH)_3_ also hinders the dissolution–recrystallisation mechanism that commonly triggers NP agglomeration, particle coarsening, and faceted growth.

An analogous investigation was carried out by Rendon-Angeles et al. [7], which focused on the one-step synthesis of Uvarovite NPs (Ca_3_Cr_2_Si_3_O_12_), in which the single-phase stability depends on the alkaline concentration of the hydrothermal media. The single-step chemical reaction associated with the Ca_3_Cr_2_Si_3_O_12_ formation proceeded in a 5 M KOH solution under hydrothermal conditions at 200 °C for 12 h, resulting in the preparation of fine agglomerates with a popcorn-like shape and sizes varying between 66 and 156 nm. A peculiar self-assembly process involving primary irregular anhedral crystals (8.05–12.25 nm) led to the formation of the Ca_3_Cr_2_Si_3_O_12_ agglomerates. The Ca(OH)_z_^n+^ and Cr(OH)_m_^n+^ formed in the reaction media due to a deficient Si^4+^ nominal content, resulting in a size decrease in the 3D popcorn-shaped agglomerates. The particle size variation led to a variation in the colour hue. At the same time, the faceted surface texture inherited from the tiny primary NPs achieved a high-reflectance near-infrared spectrum, which is vital in designing cold pigments for industrial insulating applications.

Differences in the shapes and growth mechanisms of NPs likely depend on the chemical reaction pathway between the solid precursor and the aqueous media, which was demonstrated by the study conducted by Luo and Taleb [8]. Their analysis considers the usage of TiO_2_ aggregates constituted by NPs and aims to elucidate the mechanism that controls the morphology of TiO_2_ under hydrothermal conditions in a highly concentrated NaOH medium (10 M). An increase in the reaction temperature from 100 to 200 °C caused the consumption of aggregated particles of nano-urchin-shaped TiO_2_ anatase to produce TiO_2_ nanotubes and nanobelts. The preparation of the ultimate TiO_2_ nanobelts occurred by a particular assembly mechanism promoted by the Na^+^ ions in the hydrothermal media; these ions caused the exfoliation of TiO_2_ rolled nanosheets self-assembled to produce the TiO_2_ nanotubes, and with the increase in both the temperature and reaction interval, the production of TiO_2_ nanobelts. According to their morphological features, TiO_2_ nanobelts can be used to prepare anodes for Li-ion batteries.

In addition, the characterisation of microstructural NPs is essential for the morphological study of their features (particle size, surface area, geometry, and chemical compositional analysis). The details of the NPs’ microstructures are currently revealed by transmission electron microscopy (TEM). However, additional information, such as overlapping particles, faceted particle growth, and 3D hierarchical self-assembly architectures, are complicated to analyse using TEM observations. Hence, Asano et al. [9] proposed an innovative technological approach based on scanning electron microscopy (SEM) to overcome the deficiencies of TEM observation. A peculiar holder constituted by an ultrathin Si_3_N_4_ window allowed the authors to observe CeO_2_ NPs in water in the SEM machine. This tool makes it possible to directly observe the architectural features of CeO_2_ nanoclusters synthesised under hydrothermal conditions. Improvements in new SEM devices boost their performance and versatility, allowing them to obtain high-resolution images of hydrothermally synthesised nanoparticles. Various examples, including details of particle preparation and SEM operating conditions, are discussed in detail in this paper. The methodologies derived from this work will increase the motivation of researchers to characterise NPs using this technique.

Finally, a review of the fundamentals of the oxidation process of SiC materials is published by the research group of Biscay et al. [10]. The review provides a detailed overview of the state-of-the-art in this specific research field. SiC ceramics have excellent thermomechanical and corrosion resistance and are thus suitable for high-performance applications in the aerospace and nuclear industries. Therefore, the conscientious study of the corrosion behaviour of SiC in dry and aqueous environments is required. The review presents a critical analysis of the oxidation pathways for each case, which are correlated to thermodynamic and chemical kinetics. A detailed evaluation of the models proposed for SiC oxidation under hydrothermal conditions is discussed. The last section of the review provides a comprehensive examination from an application point of view of the hydrothermal corrosion process of SiC materials. This methodology can be applied to designing SiC devices for aerospace applications; however, a dangerous scenario can arise for some critical applications, such as employing SiC to generate new nuclear reactors.

Nanotechnology is developing quickly, and significant progress is expected in developing efficient, clean, and sustainable materials. This Special Issue presents high-quality original research works and a review of advanced nanomaterials obtained via “hydrothermal synthesis” for various applications. We hope these articles will provide valuable information that motivates a generation of young scientists to continue the growth of this research field. The research in this field is booming, and significant advances in developing efficient, clean, and sustainable materials and technologies are expected.

## Data Availability

All papers comprising the Special Issue “Hydrothermal Synthesis of Nanoparticles”, including the supporting information data files, are available at https://www.mdpi.com/journal/nanomaterials/special_issues/hydrotherm_nano (accessed on 23 April 2023).
